# High-Dose Toremifene as a Promising Candidate Therapy for Hormone Receptor-Positive Metastatic Breast Cancer with Secondary Resistance to Aromatase Inhibitors

**DOI:** 10.1155/2020/7156574

**Published:** 2020-02-12

**Authors:** Atsushi Fushimi, Isao Tabei, Azusa Fuke, Tomoyoshi Okamoto, Hiroshi Takeyama

**Affiliations:** ^1^Department of Surgery, The Jikei University School of Medicine Daisan Hospital, Japan; ^2^Department of Breast & Endocrine Surgery, The Jikei University School of Medicine, Japan

## Abstract

There are currently no established second- and later-line therapies for postmenopausal women with hormone receptor-positive advanced or metastatic breast cancer. We examined the efficacy of high-dose toremifene (HD-TOR) for this patient group and whether aromatase inhibitor (AI) resistance influences HD-TOR treatment outcome. This retrospective analysis investigated the outcomes of 19 women with postmenopausal hormone-sensitive recurrent or metastatic breast cancer who received HD-TOR, defined as 120 mg daily from 2012 to 2016. The median follow-up duration was 9.67 months. The overall response rate (ORR) and clinical benefit rate (CBR) were compared between various clinical subgroups, including patients exhibiting primary or secondary AI resistance as defined by the timing of recurrence or progression. Time to treatment failure (TTF) was estimated by the Kaplan–Meier method and compared between subgroups by the log-rank test. The overall ORR was 21.1%, and the CBR was 31.6%. CBR was significantly higher for patients without liver metastasis (50% vs. 0%, *p* = 0.044). Nine cases exhibited primary and eight cases secondary AI resistance. Both ORR and CBR were higher in patients with secondary AI resistance (25% vs. 0%, *p* = 0.087; 38% vs. 11%, *p* = 0.29). The median TTF was 6.2 months in the entire AI-resistant group (*n* = 17) and was longer in the secondary resistance subgroup than in the primary resistance subgroup (8.40 vs. 4.87 months; log-rank: *p* = 0.159). High-dose TOR appears to be most effective for postmenopausal breast cancer cases with secondary resistance to AIs, cases without prior AI treatment, and cases without liver metastasis. The detailed mechanisms of AI resistance and the clinical features of responsive cases need to be further clarified to identify the best candidates for HD-TOR.

## 1. Introduction

Aromatase inhibitors (AIs) have long been the primary first-line endocrine treatment for postmenopausal women with hormone receptor-positive metastatic or locally advanced breast cancer [1, 2]. Recently, however, several prospective trials have reported that a combination of CDK 4/6 inhibitors and AIs has better efficacy as first-line endocrine therapy than AIs alone [3–5]. This change in first-line endocrine therapy has also influenced the choice of subsequent therapies. In fact, clinicians now have several options for the second- and later-line endocrine therapy, such as fulvestrant (selective estrogen receptor downregulator: SERD), AIs not used as first-line therapy, and AIs combined with CDK 4/6 inhibitors or mechanistic target of rapamycin (mTOR) inhibitors [6–8]. However, there are no firmly established second- and later-line endocrine therapies for postmenopausal women with hormone receptor-positive metastatic breast cancer. Moreover, the cost-effectiveness of these new endocrine and targeted therapies is still debated given their relatively high cost and lack of definitive evidence for superior efficacy [9, 10]. Further, these regimens have numerous side effects. Therefore, an endocrine therapy with equivalent efficacy at a lower cost is desirable.

High-dose toremifene therapy (HD-TOR) has attracted attention as an effective and relatively low-cost endocrine therapy for metastatic breast cancer. Toremifene is a selective estrogen receptor modulator (SERM) used alone as an adjuvant endocrine therapy to treat hormone receptor-positive breast cancer in Japan. The standard dose is 40 mg/day orally, and a higher dose (120 mg/day orally) is used to treat metastatic breast cancer that is unresponsive to other endocrine therapies. Although the precise mechanism underlying the anticancer efficacy of HD-TOR in cases with prior endocrine therapy failure is not yet clear, a recent report suggested the dose-dependent inhibition of the MAPK/ERK signaling pathway in addition to hormone receptor blockade [11]. Several clinical studies have found that HD-TOR is effective for metastatic breast cancer as part of “hormone rotation therapy” [12–16]. However, there are still no factors to identify cases more likely to show responsiveness to HD-TOR.

One aim of the present study is to examine the effectiveness of HD-TOR against postmenopausal hormone-sensitive progressive or recurrent breast cancer. In addition, we examined whether AI resistance influences HD-TOR efficacy because AIs are still the most frequently used first-line treatments.

## 2. Materials and Methods

A retrospective analysis was conducted to investigate the outcomes of women with postmenopausal hormone-sensitive recurrent or metastatic breast cancer who received HD-TOR (120 mg/day). We reviewed age, hormone receptor, and human epidermal growth factor receptor 2 (HER2) expression status at the latest biopsy, site(s) of metastasis at the beginning of HD-TOR therapy, therapies used before HD-TOR, and the antitumor effects of HD-TOR. The antitumor effects were judged on the basis of RECIST 1.1. The overall response rate (ORR) and clinical benefit rate (CBR) were calculated and compared between clinical subgroups defined by HER2 status, history of endocrine therapy, metastatic organ, presence of AI resistance, and type of AI resistance (primary or secondary) by Fisher's exact test.

**Figure 1:**
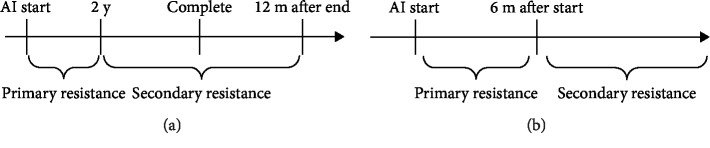
Definition of primary and secondary AI resistance in cases of AI as adjuvant therapy (a) and AI for metastatic breast cancer (b).

We classified AI resistance as primary or secondary on the basis of the following definitions. Primary resistance was defined as recurrence within 2 years after the start of adjuvant endocrine therapy with AIs or progression within 6 months from the start of AI therapy for metastatic breast cancer. Secondary resistance was defined as recurrence later than 2 years after the start of adjuvant endocrine therapy with AIs until 12 months from the end of AI adjuvant endocrine therapy or progression later than 6 months after the start of AI therapy for metastatic breast cancer (Figure 1) [6]. Time to treatment failure (TTF) was calculated using the Kaplan–Meier method and compared between clinical subgroups by the log-rank test. All data were analyzed using Stata/IC version 15.0 for Windows (StataCorp LLC, College Station, TX).

## 3. Results

In all, 21 consecutive patients (19 women and 2 men) received HD-TOR for hormone-sensitive recurrent or metastatic breast cancer at our institution from September 2012 to August 2016 (Table 1). The median follow-up duration for the 19 women included in our analysis was 9.67 months, and the median age was 70 years (range: 50–91 years). None of the four HER-2-positive patients received HD-TOR with anti HER-2 drugs. Nine patients (47.4%) demonstrated primary AI resistance, eight patients (42.1%) demonstrated secondary resistance, and two patients (10.5%) had no prior AI treatment. Seven patients had liver metastases (36.8%), 12 patients had lung metastases (63.2%), and 11 patients had bone metastases (57.9%). The seven patients with liver metastasis also had lung metastasis (5/7, 71.4%), bone metastasis (5/7, 71.4%), and soft tissue metastasis (5/7, 71.4%). One patient achieved complete remission (CR), three patients achieved partial remission (PR), two achieved long-term stable disease (SD), and 12 had progressive disease (PD). A previous study reported that HD-TOR treatment was well tolerated with no severe adverse events, although three of 43 patients experienced nausea, fatigue, hot flashes, and night sweating, which were thought to be endocrine-related symptoms [14]. In this study, as well as the previous study, no patients discontinued HD-TOR owing to intolerable side effects, such as thrombosis, and no patients reduced or discontinued the administration of HD-TOR.

The ORR was 21.1%, and the CBR was 31.6% (Table 2). The ORR and CBR were significantly higher in the subgroup of patients without prior AI treatment (ORR: 100% vs. 12%, *p* = 0.035; CBR: 100% vs. 24%, *p* = 0.088). The CBR was also significantly higher in the subgroup of patients without liver metastasis (50% vs. 0%, *p* = 0.044). The ORR and CBR tended to be higher in patients who had developed secondary resistance to AIs (ORR: 25% vs. 0%, *p* = 0.087; CBR: 38% vs. 11%, *p* = 0.29).

**Figure 2:**
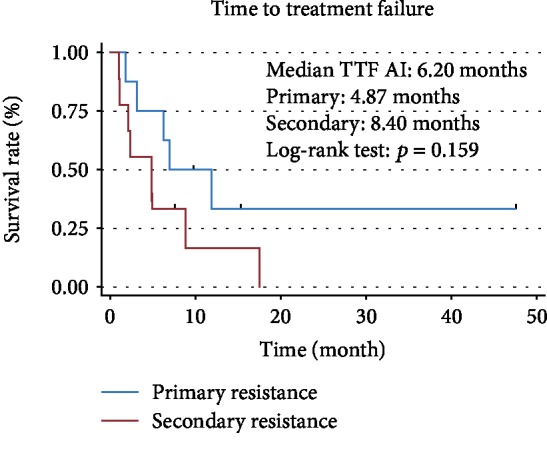
The TTF Kaplan–Meier curves for the primary and secondary AI resistance subgroups.

The 17 cases with AI resistance consisted of nine cases with primary and eight cases with secondary resistance. The primary resistance subgroup had a higher frequency of previous chemotherapy and a lower frequency of lung metastasis than the secondary resistance subgroup (chemotherapy: 78% vs. 13%; lung metastasis: 44% vs. 100%). There were no significant differences in the other factors between the primary and secondary AI resistance subgroups (Table 3).  Figure 2 shows the TTF Kaplan–Meier curves for the primary and secondary AI resistance subgroups. For the entire AI resistance group, the median TTF was 6.2 months. The median TTF of the secondary resistance subgroup was longer than that of the primary resistance subgroup (8.40 vs. 4.87 months; log-rank: *p* = 0.159).

## 4. Discussion

In this case series, HD-TOR was effective for hormone-positive metastatic breast cancer, especially in cases with secondary resistance to AIs. Resistance to AIs was defined as primary (*de novo*) or secondary (acquired) on the basis of the timing of recurrence or progression [6]. As the majority of acquired endocrine therapy-resistant breast cancer cases are ER-expressing, the loss of ER expression is very likely not the primary resistance mechanism [17]. Thus, blocking other tumor survival pathways such as PI3K/Akt/mTOR or Ras/MAPK may be required to overcome secondary resistance to AIs.

The mTOR inhibitor everolimus is a possible alternative treatment for breast cancer resistant to AIs. A randomized phase II PrE0102 trial reported that the combination of everolimus and fulvestrant doubled the progression-free survival (PFS) compared with fulvestrant alone (10.3 vs. 5.1 months) [18]. This suggests that acquired resistance to AIs is related to the PI3K/Akt/mTOR signaling pathway, a major regulator of the cell cycle [19, 20], and that a SERD or SERM plus a targeted therapy that inhibits the PI3K/Akt/mTOR pathway may have superior efficacy for breast cancer with secondary resistance to AIs.

Alternatively, MAPK signaling may contribute to AI resistance. In addition to the competitive antagonism of estrogen, HD-TOR has been suggested to suppress cancer growth through the dose-dependent inhibition of the insulin-like growth factor 1 (IGF-1)/MAPK/ERK signaling pathway [21]. AIs are known to increase serum IGF-1, thus activating the MAPK/ERK signaling pathway and inducing secondary resistance. This may explain why HD-TOR resulted in higher ORR and CBR values in patients with secondary resistance to AIs. Because HD-TOR is less expensive than other endocrine therapies with inhibitors of growth signaling pathways (Table 4), HD-TOR is a suitable candidate for breast cancer patients with secondary resistance to AIs.

Clinical studies have also found that HD-TOR is effective for metastatic breast cancer patients with relapse on AI treatment. A retrospective trial of 80 patients with AI treatment history reported ORR and CBR values of 15.0% and 45.0%, respectively, using HD-TOR [12], whereas a randomized controlled trial of 91 patients with nonsteroidal AI failure found a substantially longer median PFS with HD-TOR treatment than exemestane treatment (7.3 vs. 3.7 months; log-rank test: *p* = 0.045) [14]. However, neither of these studies distinguished the type of AI resistance (primary or secondary). The current results suggest that HD-TOR would have been even more effective in those patients with secondary resistance to AIs.

The CBR found in the current study (31.6%) is relatively low compared with previous studies, but several of these studies found a negative association between CBR and the rate of liver metastasis (Table 5) [12–16]. Thus, the low CBR may be due to a higher frequency of liver metastasis than in previous studies. The reason for lower HD-TOR efficacy in patients with liver metastasis is unknown, and there is no evidence that endocrine therapies are less effective in breast cancer cases with liver metastasis. In the current study, however, the patients with liver metastasis had a larger tumor burden than those without liver metastasis, including additional tumor sites in the lung, bone, and soft tissue. In cases with a large tumor burden, chemotherapy is preferred over endocrine therapy [22]. Therefore, changing from hormone rotation therapy to chemotherapy should be considered for cases with liver metastasis or a large tumor burden.

This study has several limitations. First, the study population was too small to evaluate HD-TOR efficacy between the known risk factor subgroups. The small study population also precluded detailed side effect evaluation. In addition, side effect comparisons were limited by the retrospective study design. However, we found no obvious side effects even after an extensive review of the medical records.

## 5. Conclusions

High-dose toremifene appears to be effective for advanced breast cancer with secondary resistance to AIs but is not effective in advanced cases with liver metastasis. Further studies are warranted to identify additional factors predictive of HD-TOR efficacy for advanced breast cancer.

## Figures and Tables

**Table 1:** Patient characteristics.

			*N*	%
Total			19	100

Age (years)			70	

Hormone receptor	ER+/PgR+		14	73.7
	ER+/PgR−		4	21.1
	ER−/PgR+		1	5.3
	ER−/PgR−		0	0.0

HER2	Positive		4	21.1
	Negative		15	78.9

History of therapy	Tamoxifen		4	21.1
	AI		17	89.5
	Trastuzumab		2	10.5
	Chemotherapy		9	47.4
	Type of chemotherapy	Anthracycline	8	42.1
		Taxane	7	36.8
		5-FU	3	15.8
		Eribulin	1	5.3
		Vinorelbine	1	5.3

Site of metastasis	Liver		7	36.8
	Lung		12	63.2
	Bone		11	57.9
	Pleura		2	10.5
	Peritoneum		1	5.3
	Soft tissue		9	47.4

AI resistance	Primary		9	47.4
	Secondary		8	42.1

Response of HD-TOR	CR		1	5.3
	PR		3	15.8
	ORR		4	21.1
	Long SD		2	10.5
	CBR		6	31.6
	SD		0	0.0
	PD		12	63.2
	NE		1	5.3

HER2: human epidermal growth factor receptor 2; AI: aromatase inhibitor; HD-TOR: high-dose toremifene; CR: complete response; PR: partial response; ORR: overall response rate; SD: stable disease; CBR: clinical benefit rate; PD: progressive disease; NE: not evaluable.

**Table 2:** Factors predictive of high-dose toremifene therapeutic efficacy.

			ORR			CBR		
		*N*	*n*	%	*p* value	*n*	%	*p* value
Total		19	4	21.1		6	31.6	

HER2	Positive	4	1	25.0	1.000	1	25.0	1.000
	Negative	15	3	20.0		5	33.3	

History of tamoxifen	Yes	4	1	25.0	1.000	2	50.0	0.557
	No	15	3	20.0		4	26.7	

History of AI	Yes	17	2	11.8	0.035	4	23.5	0.088
	No	2	2	100.0		2	100.0	

Liver metastasis	Yes	7	0	0.0	0.245	0	0.0	0.044
	No	12	4	33.3		6	50.0	

Lung metastasis	Yes	12	2	16.7	0.603	3	25.0	0.617
	No	7	2	28.6		3	42.9	

Bone metastasis	Yes	11	1	9.1	0.134	2	18.2	0.141
	No	8	3	37.5		4	50.0	

Visceral metastasis	Yes	14	2	14.3	0.272	3	21.4	0.262
	No	5	2	40.0		3	60.0	

AI resistance	Primary	9	0	0.0	0.087	1	11.1	0.294
	Secondary	8	2	25.0		3	37.5	

ORR: overall response rate; CBR: clinical benefit rate; HER2: human epidermal growth factor receptor 2; AI: aromatase inhibitor.

**Table 3:** Characteristics of primary and secondary AI resistance subgroups.

		Total (*N* = 17)	AI primary resistance (*N* = 9)		AI secondary resistance (*N* = 8)		
		*N*	*n*	%	*n*	%	
Estrogen receptor	Positive	17	9	100	8	100	NA
	Negative	0	0	0	0	0.0	

Progesterone receptor	Positive	13	6	67	7	86	0.576
	Negative	4	3	33	1	14	

HER2	Positive	3	3	33	0	0	0.206
	Negative	14	6	67	8	100	

History of therapy	Tamoxifen	3	2	22	1	13	1.000
	Chemotherapy	8	7	78	1	13	0.015
	Trastuzumab	2	2	22	0	0	0.471

Previous HT lines	One line	7	4	44	3	38	1.000
	Two lines	10	5	56	5	62	

Site of metastasis	Liver	7	4	44	3	38	1.000
	Lung	12	4	44	8	100	0.029
	Bone	10	6	67	4	50	0.637
	Pleura	2	1	11	1	13	1.000
	Peritoneum	1	1	11	0	0	1.000
	Soft tissue	7	5	56	2	25	0.335

AI: aromatase inhibitor; NA: not applicable; HER2: human epidermal growth factor receptor 2; HT: hormone therapy.

**Table 4:** Drug cost per month in each common endocrine therapy in Japan on March 2019.

Endocrine therapy	Regimen	Common side effects	Drug cost per 28 days (generic drug)
TAM	Tamoxifen 20 mg/day	Hot flash, thrombosis	7,294 JPY (1,590 JPY)
ANA	Anastrozole 1 mg/day	Hot flash, arthritis, osteoporosis	11,301 JPY (4,312 JPY)
FUL	Fulvestrant 500 mg/4 weeks	Pain at injection site	101,584 JPY
EXE+mTOR	Exemestane 25 mg/day Everolimus 10 mg/day	Interstitial pneumonitis, stomatitis	593,743 JPY (589,123 JPY)
LET+PAL	Letrozole 2.5 mg/day Palbociclib 125 mg/day, 21 days	Bone marrow suppression, stomatitis	487,931 JPY (477,694 JPY)
FUL+PAL	Fulvestrant 500 mg/4 weeks Palbociclib 125 mg/day, 21 days	Bone marrow suppression, stomatitis	575,350 JPY
HD-TOR	Toremifene 120 mg/day	Hot flash, thrombosis	25,368 JPY (13,079 JPY)

HD-TOR: high-dose toremifene.

**Table 5:** Associations between liver metastasis and HD-TOR clinical benefit rate.

Study	Cases (*n*)	Liver metastasis (*n*) (%)	ORR (%)	CBR (%)	Year	Reference
Retrospective	80	10 (12.5)	15.0	45.0	2010	Yamamoto et al. [12]
Retrospective	13	3 (23.0)	7.7	46.2	2012	Sawaki et al. [16]
Retrospective	21	2 (9.5)	21.1	63.2	2013	Koike et al. [13]
Prospective	46 (HD-TOR arm)	7 (15.2)	10.8	43.2	2013	Yamamoto et al. [14]
Retrospective	85	17 (20.0)	21.2	41.2	2014	Ishizuna et al. [15]
Retrospective	19	7 (36.8)	21.1	31.6	2019	This study

HD-TOR: high-dose toremifene; ORR: overall response rate; CBR: clinical benefit rate.

## Data Availability

The excel data used to support the findings of this study are available from the corresponding author upon request.
